# Computational vaccinology and the ICoVax 2012 workshop

**DOI:** 10.1186/1471-2105-14-S4-I1

**Published:** 2013-03-08

**Authors:** Yongqun He, Zhiwei Cao, Anne S De Groot, Vladimir Brusic, Christian Schönbach, Nikolai Petrovsky

**Affiliations:** 1 Unit for Laboratory Animal Medicine, Department of Microbiology and Immunology, and Center for Computational Medicine and Bioinformatics, University of Michigan Medical School, Ann Arbor, MI 48109, USA; 2 Department of Biomedical Engineering, College Life Science and Technology, Tongji University, Shanghai, 200092, China; 3 EpiVax, Inc., Providence, RI 02903, USA; 4 Institute for Immunology and Informatics, University of Rhode Island, Providence, RI 02903, USA; 5 Cancer Vaccine Center, Dana-Farber Cancer Institute, Boston, MA 02115, USA; 6 Department of Bioscience and Bioinformatics, Kyushu Institute of Technology, Fukuoka 820-8502, Japan; 7 Biomedical Informatics Research and Development Center (BMIRC), Kyushu Institute of Technology, Fukuoka 820-8502, Japan; 8 Flinders Medical Centre, Bedford Park, SA 5042, Australia

## Abstract

Computational vaccinology or vaccine informatics is an interdisciplinary field that addresses scientific and clinical questions in vaccinology using computational and informatics approaches. Computational vaccinology overlaps with many other fields such as immunoinformatics, reverse vaccinology, postlicensure vaccine research, vaccinomics, literature mining, and systems vaccinology. The second ISV Pre-conference Computational Vaccinology Workshop (ICoVax 2012) was held on October 13, 2013 in Shanghai, China. A number of topics were presented in the workshop, including allergen predictions, prediction of linear T cell epitopes and functional conformational epitopes, prediction of protein-ligand binding regions, vaccine design using reverse vaccinology, and case studies in computational vaccinology. Although a significant progress has been made to date, a number of challenges still exist in the field. This Editorial provides a list of major challenges for the future of computational vaccinology and identifies developing themes that will expand and evolve over the next few years.

## Introduction and background

Development of effective vaccines for some of the major infectious diseases contributed to dramatic improvements in public health worldwide over the past 100 years. In the post-genomics and information era, the application of computational tools to vaccine research and development (R&D) has contributed to progress in the development of new vaccines. Computational vaccinology is a branch of vaccinology that is focused on solving scientific questions in vaccinology using computer-driven algorithms. This interdisciplinary field of research spans computer science, mathematics, statistics, molecular biology, microbiology, immunology, and vaccinology. Computational vaccinology has also been called vaccine informatics [1]. The two terms are used interchangeably to represent the same concept at this point in the history of vaccinology.

### Role of immunoinformatics

Computational vaccinology or vaccine informatics is closely related to immunoinformatics. Many immunoinformatics methods have been developed since 1980s to predict T-cell immune epitopes and B-cell functional neutralizing or cross-reactive epitopes [2]. These epitopes are useful for the development of diagnostic tests, for the development and design of vaccines, and for characterizing targets of immune responses to vaccines and infections. A large number of computational algorithms and software programs have been developed for immune epitope prediction. In general, T-cell immune epitope prediction can be successful. However, functional B-cell antibody epitopes prediction remains a challenge, especially conformational epitopes [3].

### Reverse vaccinology

Computational vaccinology has also contributed to the "reverse vaccinology" approach to vaccine development. Reverse vaccinology starts with bioinformatics analysis of genome or proteome sequences of pathogens [4] and proceeds "backwards" to identifying critical antigens, rather than beginning with selection of a single antigen and moving forward with iterative testing. This comprehensive initial screening of genome sequences enables the selection of antigen candidates that are highly likely to be relevant for vaccine development. An example of successful reverse vaccinology is the Meningitis B vaccine developed by Rino Rappuoli's group (Novartis). This vaccine is under review by the European Medicines Agency. Approval of this vaccine would signal the maturation of computational biology field. A number of other vaccines are in development, using reverse vaccinology have also been reported [1,5].

### Vaccinomics and systems vaccinology

Computational vaccinology also relates to "omics" and systems biology. Specifically, the term "vaccinomics" or "systems vaccinology" was coined to represent a new field that integrates immunogenetics and immunogenomics with "omics-based" systems biology and immune profiling methods for the better development of next-generation of vaccines and expansion of personalized medicine studies [6]. Genome-wide association studies (GWAS) have shown associations of HLA alleles at various degrees with persistent infection with hepatitis B virus [7], replication of HIV-1 [8] and response to MRKAd5 HIV-1 vaccine [9]. With the growth of personal genome and SNP data, GWAS are expected to delineate host susceptibility factors in vaccine responses on a more global scale including populations that are currently underrepresented in HapMap. Immunoinformatics provides a fundamental set of tools in the emerging field of systems immunology [10]. Similarly, computational vaccinology is critical to the advancement of systems vaccinology that must consider both pathogen and host variability.

### Literature mining

Literature mining can be considered as a tool within the scope of systems vaccinology. Currently, there are over 300,000 vaccine-related peer-reviewed articles cited in the PubMed literature database [11]. The number of vaccine-related articles in the database is increasing exponentially [1]. Vaccine-related literature mining studies have been reported in vaccine design [12], vaccine-pathogen gene interactions [13], and vaccine-associated host gene response discovery [14-16], among others.

### Postlicensure vaccine research

Postlicensure vaccine usage and safety surveillance is another field that has benefitted from computational vaccinology [1]. For example, informatics methods have been used to develop the U.S. Vaccine Adverse Event Reporting System (VAERS) in the U.S. [17] and the Vaccine Safety Datalink (VSD) [18] to monitor vaccine safety. Computerized immunization information systems have been developed to accurately track vaccination history and support postlicensure vaccine research and safety surveillance [19].

### Advances in computational vaccinology

Mathematical modeling of various aspects of infectious diseases [20] plays an important role in the formation of immunization program policies [21]. Computational vaccinology is contributing to the field of "artificial immune system". Further advances in this field may reduce the need for testing vaccines in animals. A proof of concept was demonstrated in a pre-clinical study of vaccination that prevents the development of mammary tumors in mice [22]. In that study, agent-based mathematical models were used to determine protective vaccination schedules.

Internet resources for vaccines are abound, however many of these are focused on the clinical uses and regulatory issues related to vaccines [1]. The Vaccine Investigation and Online Information Network (VIOLIN) a web-based comprehensive vaccine database and analysis system primarily targeted for vaccine researchers [23]. To promote vaccine data standardization, integration, and computer-assisted reasoning, the collaborative, a collaborative effort to develop a community-based Vaccine Ontology (VO) has recently been initiated [24]. VO has been shown to support vaccine data classification [25] and literature mining [13,14,16].

Advances in computational vaccinology are presented in a range of scientific meetings. For example, the International Conference on Bioinformatics (InCoB), an Asia Pacific scientific conference on bioinformatics [26], frequently reports selected computational vaccinology advances. The International Conference on Artificial Immune Systems (ICARIS) provides a venue for computational vaccinologists who are interested in systems-based approaches to vaccine research. The Immunoinformatics and Computational Immunology Workshop (ICIW), held in conjunction with the ACM International Conference on Bioinformatics and Computational Biology, aims to bridge the immunology, bioinformatics and computer science [27].

To improve communications between computational vaccinologists and more traditional vaccine researchers, the first Computational Vaccinology Workshop was held in 2011 at Seattle, Washington, USA, prior to the 5^th ^Vaccine & ISV Annual Global Congress. The annual Vaccine & ISV Annual Global Congress, co-organized by the journal Vaccine and the International Society for Vaccines (ISV), is the largest non-commercial conference in the vaccine field and attended by vaccine experts and researchers around the world. The second ISV Pre-conference Computational Vaccinology Workshop (ICoVax 2012) was held on October 13, 2012, in Shanghai, China [28]. This workshop occurred one day prior to the sixth Vaccine & ISV Annual Global Congress.

### ICoVax 2012 workshop

ICoVax aspires to become an international forum for researchers to report, summarize, and discuss the most recent developments and ideas in the emerging areas of computational vaccinology and vaccine informatics, and to improve our understanding of basic vaccine mechanisms and the application of computational tools to vaccine development.

The conference was well attended, and fourteen paper submissions were received and reviewed. Six full-length papers, five abstracts, and one software demonstration were accepted for presentation at the workshop. Five full-length papers were selected for extension and accepted for publication in a special issue in the journal *BMC Bioinformatics*. These papers cover the development of new programs and the applications of existing programs.

#### Software program development

In the workshop, Wang *et al*. introduced a comprehensive evaluation and optimization of sequence-, motif- and SVM-based computational prediction approaches for allergens [29]. First, the researchers collected a comprehensive dataset of 989 known allergens and an even larger number of putative non-allergens. The prediction approaches were then integrated with this data in a new web-based application "proAP" that enhances allergen search and prediction.

Xiang and He introduced the web-based reverse vaccinology software program Vaxign [30] for genomics-based prediction of vaccine targets, and its application in predicting vaccine candidates for herpes simplex virus (HSV) types 1 and 2 (HSV-1 and HSV-2) [31]. The HSV-1 protein UL26.5 was predicted to be an adhesin and a promising candidate for HSV vaccine development. This study provided an example of Vaxign-based viral vaccine design. A software demonstration of Vaxign was also conducted in the workshop.

A major informatics software development topic is 3D protein structure-based predictions and their applications. In the workshop, Dr. Zhiwei Cao introduced available tools in conformational epitope prediction. She also demonstrated the protein antigen spatial epitope prediction web server (SEPPA), a conformational epitope prediction tool developed by her group [32]. Lo *et al*. presented the development of a new method (named "CE-KEG") that combines an energy profile for surface residues with the frequency of each geometrically related amino acid residue pair to identify possible conformational epitopes (CEs) in an antigenic protein [33]. This new program improves the CE identification in immunological studies and supports synthetic vaccine design. Lo *et al*. presented a new approach called PLB-SAVE that uses only geometrical features of proteins and obtained a good overall performance for the prediction of protein-ligand binding regions [34]. PLV-SAVE outperforms two other well-known prediction systems with high accuracy rates and efficient computational time. PLB-SAVE can be applied to predict carbohydrate-antibody interactions for further design and development of carbohydrate-based vaccines.

Three additional software programs were introduced in the workshop as short talks and/or posters: the software program MetaMHCIIpan (a consensus approach for pan-specific HLA-DR binding predictions) [35], SAROTUP 2.0 (a suite of web tools for finding potential target-unrelated peptides from phage display data) [36], and the iVAX web-based vaccine design program [37].

#### Applications using available software programs

In this workshop, Chen *et al*. presented the use of bioinformatics tools including the SEPPA spatial epitope prediction program to predict possible cross-reactive spatial epitopes from norovirus capsid proteins [38]. Two common epitope regions on the capsid sequences of Group I and II norovirus genogroups, and an exclusive epitope region in Group II genogroup were identified. In addition to this full paper presentation, one short talk and one poster presentation were made, focusing on application of EpiMatrix program from EpiVax, Inc. The short talk by Wei et al. focused on the identification of promiscuous CD4+ T cell epitopes contained within the sequence of the polyprotein of hand, foot and mouth disease (HFMD) virus strain EV71 [39]. The most dominant epitope identified in this study is highly conserved in polioviruses and other enterovirus species; information presented in this short talk was recently published as a paper [40]. Gustiananda *et al*. analyzed T-cell epitopes from the envelope proteins of Dengue virus vaccine strain ChimeriVax and compared them to those from circulating viral strains in Indonesia [41]. Potential cross-reactivity was identified which might explain the efficacy (and lack of efficacy) of ChimeriVax against circulating strains of Dengue virus.

### Future challenges and prospects

The studies presented in the ICoVax 2012 workshop demonstrate the productive efforts in developing and applying state-of-the-art computational and informatics methods in the field of computational vaccinology. While significant progress has been achieved to date, many challenges still exist in this field.

#### (1) Challenges in vaccine design

(a) Specific and sensitive prediction of functional B cell antibody epitopes, in particular the conformational B cell epitopes. A conformational (or discontinuous) epitope is an epitope whose residues are distantly located in the protein sequence but are in physical proximity in the folded protein. Approximately 90% of all antibodies are raised against conformational B-cell antibody epitopes. Since many vaccines require strong antibody responses, conformational B-cell antibody epitope prediction becomes critical for rational vaccine design. While the prediction of non-conformational or linear epitopes has been proven more difficult than the prediction of T cell epitopes [42], the prediction of conformational B cell functional epitopes is more challenging. The native 3-dimensional (3D) protein structure is crucial to the prediction of conformational epitopes. When a 3D protein structure is unknown, the prediction of the 3D structure often becomes a prerequisite for conformational epitope prediction. The relations between conformational epitopes and 3D protein structure then require further clarification.

(b) Cancer vaccine design. Since the 1950s the development of effective cancer vaccines continues to be a challenge. However, a number of clinical trials have been delivering promising results that reflect the importance of broad T cell responses to a range of T cell epitopes [43]. Further success of cancer vaccine development mostly requires the identification of antigens that are unique to cancers, and combining the antigens with an effective adjuvant and biologics that block regulatory T cell responses such as anti-CTLA4. This is one area of translational computational immunology research that is progressing rapidly to the clinic [44].

(c) Allergy vaccine design. One means of developing allergy vaccines is to modify allergen molecules with an aim to reduce both IgE and allergen-specific T cell epitope responses [45]. Alternatively, T cell epitopes can be administered subcutaneously, leading to the induction of antigen-specific tolerance. One company has mapped all of the sequences of the cat dander allergen Fel d 1 using immunoinformatics tools and successfully translated this information into clinical use [46]. Additional vaccines, developed using immunoinformatics tools, are in clinical development.

(d) Autoimmune disease vaccine design. Examples of autoimmune diseases include rheumatoid arthritis, eczema, and multiple sclerosis. This field is ripe for future applications of immunoinformatics tools, since in many cases the autoantigens are known.

#### (2) Challenges in basic vaccine mechanism studies

(a) Better understanding of fundamental vaccine-induced protective immunity. Vaccine-induced protective immunity may be similar to or different from the pathogen-induced host immunity. To differentiate these two types of immune responses is critical to understand protective immune mechanism.

(b) Identification of immune correlates of protection and gene markers that predict protective immunity. Until now, the immune correlates of protection are unknown for most pathogens. Sufficient and necessary gene markers for predicting protective immunity are not available for most pathogens, either. The "omics-based" systems vaccinology approach has started to provide a powerful way to detect immune correlates and gene markers of protection for pathogens including influenza virus [47] and Yellow fever virus [48,49].

#### (3) Challenges in post-licensure computational immunology

An electronic health record (EHR) is a collection of patient health information including clinical date. A large amount of EHR data from post-licensure vaccine usage is also available in different immunization registry systems, vaccine adverse events reporting system, and clinical information systems. Processing and analysis of the vaccine-related EHR datasets offers promises of gaining valuable knowledge but it also presents a challenge. Many EHR data are unstructured and thus require some kind of natural language processing (NLP) to dissect them into structured data. Ontological representation and modeling of EHR data can help better classification and analysis of the vaccine EHR data [50]. New statistical methods are needed to handle the high throughput EHR data.

#### (4) Challenges in vaccine databases and data integration

Web-based comprehensive databases are needed to collect various aspects of vaccine-related data, such as host gene markers predicting vaccine efficacy and critical pathways of vaccine-induced immune protection mechanisms. Such databases are not yet available. While the community-based Vaccine Ontology aims to support better vaccine data exchange and automated reasoning, more demonstrations are needed to show the potentials.

#### (5) Challenges in development of new algorithms and software programs

Development of novel mathematical, statistical, and computational algorithms and software programs has been a major task of computational vaccinology. New challenges and questions require the development and use of more innovative algorithms and tools than currently available.

These challenges pose new opportunities for researchers in computational vaccinology. The field of computational vaccinology has attracted some of the most talented researchers from different research areas. We look forward to more exciting findings and tools coming out in the near future to support the important vaccine research and development.

## Competing interests

The authors were organizing committee members of ICoVax2012. YH and ZC served as co-chairs. ASDG is the founder and majority shareholder at EpiVax, Inc., a privately owned vaccine design company located in Providence, Rhode Island, USA. ASDG is also a faculty member at the University of Rhode Islands Institute for Immunology and Informatics. This author acknowledges that there is a potential conflict of interest related to her relationship with EpiVax and attest that the work contained in this research report is free of any bias that might be associated with the commercial goals of the company. All other authors declare they have no other conflict of interest.

## Authors' contributions

YH was the lead editor. ZC, ASDG, VB, CS and NP contributed as co-editors to this special issue.

## Declarations

This article has been published as part of *BMC Bioinformatics *Volume 14 Supplement 4, 2013: Special Issue on Computational Vaccinology. The full contents of the supplement are available online at http://www.biomedcentral.com/bmcbioinformatics/supplements/14/S4
